# Experimental study of camptothecin combined with drug-eluting bead transarterial chemoembolization in the rabbit VX2 liver tumor model

**DOI:** 10.3389/fonc.2022.906971

**Published:** 2022-10-10

**Authors:** Fanguang Meng, Yuyao Li, Qian Liu, Liwei Sun, Hankang Wang, Xiaodong Li, Guijie Li, Feng Chen

**Affiliations:** ^1^ Department of Radiology, The First Affiliated Hospital of Shandong First Medical University & Shandong Provincial Qianfoshan Hospital, Shandong Medicine and Health Key Laboratory of Abdominal Medicine Imaging, Jinan, China; ^2^ Shandong First Medical University & Shandong Academy of Medical Sciences, Jinan, China; ^3^ Undergraduate, Department of Clinical Medicine, Faculty of Clinical and Basic Medicine, Shandong First Medical University & Shandong Academy of Medical Sciences, Jinan, China; ^4^ Department of Radiology, The Second Affiliated Hospital of Zhejiang University School of Medicine, Zhejiang, China

**Keywords:** hepatocellular carcinoma, DEB-TACE, chemoresistant, NRF2, camptothecin, chemosensitization

## Abstract

Drug-eluting bead transarterial chemoembolization (DEB-TACE) has been widely used in the treatment of liver cancer; however, the utilization rate of chemotherapeutic drugs after embolization is low. Chemotherapy resistance mediated by high nuclear factor E2-related factor 2 (NRF2) expression limits DEB-TACE efficacy. Camptothecin (CPT), an NRF2 inhibitor, exerts chemosensitizing effects. We designed a controlled experiment to determine the efficacy and feasibility of DEB-TACE combined with CPT for the treatment of rabbit VX2 hepatoma. DEB-TACE activated NRF2 expression in the tumor region. NRF2 activation could be inhibited by the combined use of CPT. After DEB-TACE alone, the tumor necrosis was incomplete, there were still highly active tumor residues, and the apparent diffusion coefficient (ADC) value, which was negatively correlated with tumor activity observed by magnetic resonance imaging, remained low. After DEB-TACE combined with CPT, the relative necrosis of the tumor was more complete, the ADC value was higher, and the ADC change was greater. The single application of CPT did not result in evident liver function and physical burden to the rabbits. The combined use of CPT and DEB-TACE did not significantly increase DEB-TACE imaging of liver function and body. In conclusion, CPT can also inhibit high NRF2 expression after DEB-TACE treatment. Combining CPT with DEB-TACE can improve the sensitivity of DEB-TACE in the treatment of VX2 tumors, improve the therapeutic effect, and has no evident toxic and side effects. This study explored the methods for enhancing the efficacy of DEB-TACE in liver cancer from a new perspective and performed model experiments, which provided a theoretical basis for future clinical treatment.

## Introduction

Drug-eluting bead transcatheter arterial chemoembolization (DEB-TACE) has been widely used in the treatment of bulky and hypovascular liver cancer and has achieved good curative effect (1–3). DEB-TACE improves the safety of embolization by preventing off-target embolization and reducing the side effects of embolization therapy (4). Simultaneously, complete and lasting embolization further blocked the blood supply to the local tumor. However, studies have shown that the absorption of antitumor drugs by liver cancer cells is low, and only 0.6% of antitumor drugs can kill tumor cells (5, 6).

As a regulator of oxidative stress, nuclear factor E2-related factor 2 (NRF2) mediates chemoresistance in tumor cells (7, 8). Factors such as hypermetabolism of tumors, cytotoxic effects of chemotherapeutic drugs, and blockade of tumor blood vessels activate local oxidative stress, which increases reactive oxygen species (ROS) levels (9). High concentrations of ROS can directly induce oxidative damage to DNA, proteins, and lipids in tumor cells, which in turn induces tumor cell death (7, 10). However, upon stimulation by high levels of ROS or electrophiles, NRF2 initiates the expression of a series of anti-inflammatory, antioxidant, phase II detoxification enzymes and drug transporter downstream of antioxidant response elements (AREs). NRF2 protects tumor cells from the toxic effects of drugs and ROS (11–13).

Camptothecin (CPT) and its derivatives are clinically used as DNA topoisomerase I (TOP1) inhibitors for the treatment of ovarian, lung, and colorectal cancers (14). Our previous studies showed that lower effective doses (micromolar dose levels) of CPT can inhibit the transcriptional activity of NRF2 in hepatoma cells and tumor-bearing mice. CPT combined with antitumor drugs (arsenic trioxide, doxorubicin, fluorouracil) can significantly inhibit the proliferation, invasion, metastasis, and angiogenesis of liver cancer cells, promote apoptosis of liver cancer cells, and improve the chemotherapy sensitivity of liver cancer cells (8, 15).

The low utilization rate of chemotherapy after DEB-TACE was exacerbated by the high tumor expression of NRF2. In vitro and in vivo experiments in mice have demonstrated the feasibility of CPT for inhibiting NRF2 expression and enhancing chemotherapeutic drug sensitivity (8). In the combined application of DEB-TACE and CPT, CPT is used to inhibit the activation of NRF2 after DEB-TACE to achieve chemosensitization and improve the utilization rate of loaded drugs and the therapeutic effect of DEB-TACE. However, to date, no studies related to this idea have been reported. To establish a rabbit VX2 liver cancer model, we combined DEB-TACE with CPT, preliminarily explored the feasibility and efficacy of this treatment method, and determined a new method to improve the efficacy of DEB-TACE for liver cancer.

## Materials and methods

### Chemicals, reagents, and equipment

**Figure 1:**
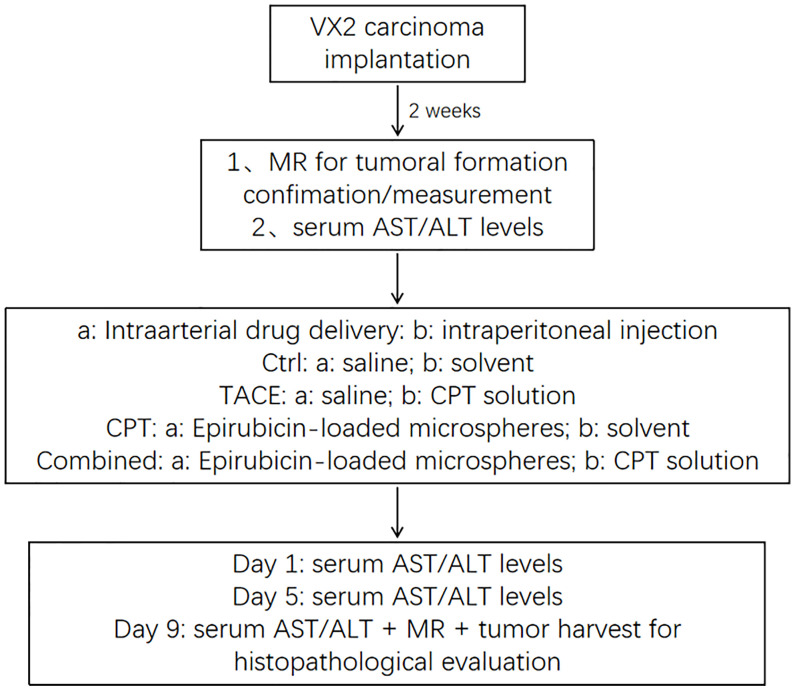
Overview of the experimental design.

**Figure 1** shows the basic steps of the experiment. Prior to the formal experiment, we conducted a preliminary experiment to improve the experimental details. Epirubicin (EPI) and CPT were purchased from Selleck (Shanghai, China). CalliSpheres Beads (100–300 μm) were obtained from Hengrui Pharmaceuticals Co., Ltd. (Jiangsu, China). CalliSpheres Beads loaded EPI in a common manner (3, 16). CPT was dissolved in a solvent comprising dimethyl sulfoxide, hydroxymethyl cellulose, and Tween-80 (17). NRF2, HO-1 and NQO1 antibodies were obtained from Servicebio (Wuhan, China). PBS buffer, TBST buffer, crystal violet staining solution, H&E staining and immunofluorescence (IF) staining reagents were received from Servicebio (Wuhan, China). SDS-PAGE Gel Preparation Kit, protein maker, HRP-labeled Goat Anti-Rat IgG were provided by EpiZyme (Shanghai, China).

### Liver tumor model

**Figure 2:**
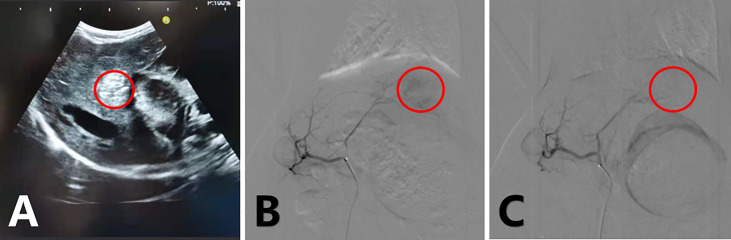
Ultrasound-guided tumor implantation, hyperechoic coda in the liver **(A)**; arterial angiography of a rabbit VX2 liver tumor before treatment **(B)** and immediately after treatment **(C)** with epirubicin-loaded CB. Tumor staining disappeared after embolization.

New Zealand White rabbits (both sexes, 2.5–3 kg in weight) were used in this study. VX2 carcinoma was planted in the muscles of young rabbits’ limbs to maintain a continuous supply of tumors (18, 19). After a 12-h fast, rabbits were anesthetized with an intramuscular injection of 2 mg/kg xylazine hydrochloride (Sumianxin II, Jilin, China), and an intravenous injection of 30 mg/kg sodium pentobarbital was administered (20). Under ultrasound guidance (**Figure 2A**), an 18-G needle was used to puncture the parenchyma of the left lobe of the rabbit liver through the subxiphoid region at a depth of approximately 1.5 cm. A 1 mm3 VX2 tumor fragment was placed into the needle and sealed with a Gelfoam strip, and the needle core was returned (19).

### Magnetic resonance imaging (MRI) scan

After a 2-week growth period, the tumor should grow to its target state. Magnetic resonance imaging (MRI) included T2-weighted images (T2), and diffusion-weighted imaging (DWI) (GE, USA) (T2 sequence: repetition time [TR], 5200; echo time [TE], 88.7; layer thickness, 2 mm; NEX, 2.0; DWI sequence: TR, 4500; TE, 81.6; layer thickness, 2 mm; NEX, 2.0; b = 800) was performed to measure tumor size and activity. A single 2-cm-diameter tumor was confirmed (21). Nine days after the surgery, MRI scans with the same parameters were performed again (22). All images were sent to a workstation for post-processing. The largest cross-sectional area of the tumor was marked, which was used to assess tumor growth, the apparent diffusion coefficient (ADC) value in this area was determined, and the change in ADC (ΔADC, postoperative ADC − preoperative ADC) was calculated and used to assess tumor activity.

### Experimental groups

All rabbits were provided two routes of administration: intra-arterial (IA) drug delivery and intraperitoneal (IP) injection. We completed the modeling of 32 rabbit VX2 liver cancer models and divided them into four groups randomly: Ctrl group (5 ml normal saline [IA drug delivery] and 5 ml solvent [IP injection, three times a week {TIW}], n = 8), CPT group (3 m/kg CPT [IP injection, TIW], 5 ml normal saline [IA drug delivery], n = 8), TACE group (0.3 ml EPI-loaded CalliSpheres Beads [IA drug delivery], 5 ml solvent [IP injection, TIW], n = 8), and Combined group (3 m/kg CPT [IP injection, TIW) and 0.3 ml EPI-loaded CalliSpheres Beads [IA injection], n = 8).

### Arterial chemotherapy and intraperitoneal administration

Hepatic arterial chemoembolization was performed on all tumor-bearing rabbits by two experienced interventional physicians using digital subtraction arteriography (Siemens Medical Solutions, Munich, Germany). All animals were operated on according to the same procedure. Before treatment, the animals were sedated and anesthetized, the skin was prepared and fixed, and the femoral artery was isolated following the principle of aseptic surgery. Subsequently, 2% lidocaine was locally infiltrated and the femoral artery was dilated; the femoral artery was punctured using the Seldinger method, and a 4-F sheath was placed. Thereafter, a 2.7-F coaxial microcatheter system (Terumo, Tokyo, Japan) was used to select the celiac artery for angiography to identify the hepatic artery (18–21, 23). Next, we selected the hepatic artery and tumor-feeding artery (generally the left branch of the hepatic artery) for the angiography. After angiography confirmed the complete blood supply to the arteries of the tumor, the four groups were provided scheduled intervention, and the tumor blood supply was reassessed by angiography after treatment (**Figures 2B, C**). The sutures were pre-positioned, all catheters with sheaths were removed, and the wound was closed. All rabbits were administered penicillin for 3 days postoperatively. Intraperitoneal injections were administered to all tumor-bearing rabbits on the day of the operation and 3 and 6 days after treatment. The CPT and Combined groups were injected with 5 ml of CPT solution, and the Ctrl and TACE groups were injected with 5 ml of solvent.

### Serum biochemical analyses

For the analysis of serum biochemistry using a standard protocol, whole blood was centrifuged at 3500 rpm for 10 min and serum in the supernatant was tested using a biochemical auto-analyzer (Type 800; Chemray, China). Serum levels of ALT and AST were used to test for liver function.

### Sample collection

Plasma samples were collected from the marginal ear vessels of rabbits (3 days preoperatively and 3, 6, and 9 days after treatment). The serum was then isolated and stored at –80°C before being tested for acute liver damage.

After the MRI scan, all animals in the four groups were euthanized on day 9. Collected tumor samples were stored in a formalin fixative solution at 4°C. Tissue sections were stained with hematoxylin and eosin (Sigma-Aldrich) for tumor necrosis, and immunohistochemical staining was performed using an NRF2 rabbit polyclonal antibody (dilution: 1:1000; Servicebio, Wuhan, China). The target NRF2 was positively expressed according to cytoplasmic tan staining.

### Western blot analysis

Tumors were smashed in the RIPA buffer with 1 mM PMSF on ice. The cells were treated for 48 h and were lysed. Next, tissue and cells were centrifuged, and supernatants were mixed with loading buffer. The samples were loaded onto the SDS-PAGE gel, and electrophoresis of transmembrane was performed. Proteins were transferred to a membrane (Servicebio, China), and target membranes were covered with blocking buffer followed by incubation with the appropriate antibody overnight. Thereafter, the membranes were incubated with HRP-labeled goat anti-rabbit IgG and HRP-labeled goat anti-mouse IgG. Finally, membranes were covered with ECL substrate and scanned. Protein expression was represented by the gray assay of targeted membrane.

### Immunohistochemical and H&E staining

Nine days post first injection, paraffin sections of tumors were subjected to Immunohistochemical and H&E staining. The sections were incubated with EDTA antigen repair buffer (pH 9.0) to perform the antigen retrieval. Then, sections were blocked with BSA for 30 min and covered with appropriate antibody at 4°C, overnight. Next, the sections were treated with secondary antibodies. In IHC staining, the sections were incubated with HRP-conjugated secondary antibody, and then were added with DAB substrate. The hematoxylin was used to perform the nucleus counterstaining. Finally, the cell nucleus was blue, and the positive expression of the targeted protein was brownish yellow. Target NRF2 quantification for each sample was determined by a pathologist blinded to clinical and the cytoplasmic staining intensity was graded as 0 (no staining), 1 (weak), 2 (moderate), 3 (strong), and used in the formula: (percentage of weak intensity ×1) + (percentage of moderate intensity ×2) + (percentage of strong intensity ×3) = H- score (24).

H&E staining was performed according to the specifications of the H&E staining kit. Briefly, following deparaffinization and rehydration, hematoxylin was added to the sections for 5 min. Thereafter sections were covered with 1% acid ethanol reagent for 5 s. The sample was then incubated with bluing agent to enable bluing of the samples, followed by incubation in eosin solution for 10 min. Finally, the sections were dehydrated and fixed with neutral balsam. Images were obtained under an optical microscope, and showed blue nuclear and pink cytoplasmic material.

### Real time PCR

The animal tissue RNA extraction kit (Servicebio, China) was used to extract total RNA from tumor tissues according to the manufacturer’s protocol. Then, 10 μl RNA was used for c-DNA synthesis using SweScript RT I First Strand cDNA Synthesis Kit (Servicebio, China), in accordance with the manufacturer’s instructions. The RT-PCR was performed using BioRad Real-Time PCR cycler and reported by the 2−ΔΔCT method. Glyceraldehyde 3-phosphate dehydrogenase (GAPDH) was used as an internal control. The primers for the target gene were used as follows, GCLM (F): 5′-AGACGGGGAACCTGCTCAAC-3′, GCLM (R): 5′-GACATCTGGAAACTCCCTCACC-3′, HO-1 (F): 5′-GGTGACTGCCGAGGGTTTTA-3′, HO-1 (R): 5′-GTTGTGCTCAATCTCCTCCTCC-3′, AKR1C1 (F): 5′-GCCTTCAGAGGACATGAAAGTCA-3′, AKR1C1 (R): 5′-AATCAGAAGAGAGGTGCCCA-3′.

### Statistical analyses

Statistical analyses and graph creation were performed using the GraphPad Prism 8.00 software (GraphPad Software, La Jolla, CA, USA). When measurements were taken, the data are expressed as mean ± standard deviation. The two datasets were compared using an independent-sample t-test. The means between groups were statistically compared using one-way analysis of variance. Statistical significance was set as P < 0.05.

## Results

### Successful modeling and completion of experimental operations

The baseline tumor sizes before treatment were 123.9 mm2 ± 19.1, 133.4 mm2 ± 21.2, 136.8 mm2 ± 20.4, and 128.6 mm2 ± 18.7 in the Ctrl, TACE, CPT, and Combined groups, respectively. Thus, tumor sizes were evenly distributed among the groups (P =0.647). All procedures were technically successful, and the planned doses of the drugs were fully delivered. Except for one rabbit in group A who died prematurely due to lung metastases, all rabbits survived the entire experimental period and were included in all procedures. Tumor and blood samples were successfully collected.

### CPT inhibited the expression and function of NRF2 after DEB-TACE

**Figure 3:**
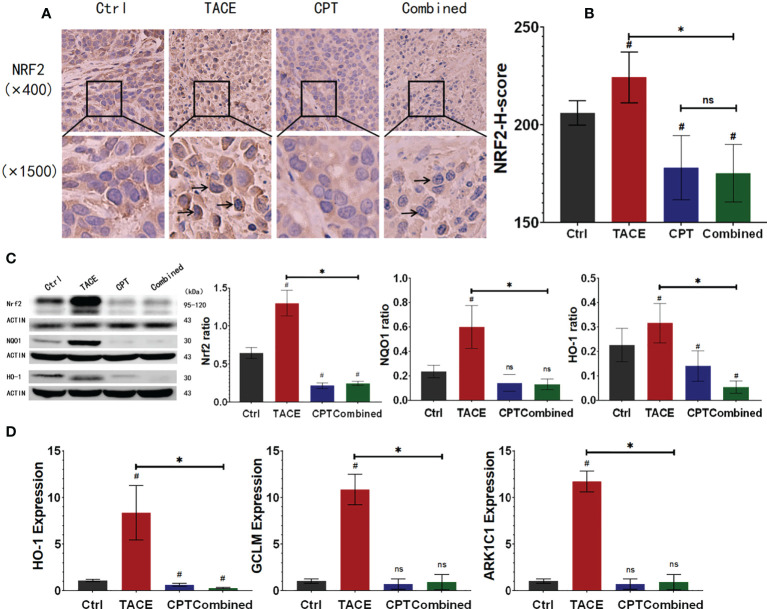
Camptothecin (CPT) inhibits the expression and functional activity of nuclear factor E2-related factor 2 (NRF2). **(A, B)** After DEB-TACE, the expression of NRF2 in the tumor area increased; following the addition of CPT, the expression of NRF2 in the Ctrl and Combined groups decreased to a lower level. **(A)** More obvious nuclear translocation of NRF2 in the TACE group, and lighter staining in the nucleus of the combined group could be observed. **(C)** The protein expression of NRF2, NQO1, and HO-1 in tumor tissue. **(D)** The RNA expression and assay of HO-1, GCLM, and ARK1C1 by qPCR. ^*^ *P* < 0.05, ^#^ *P* < 0.05, ns *P* > 0.05.

To examine the effects of DEB-TACE and CPT on NRF2 regulation in the tumors, we performed western blotting and IHC staining on tumor specimens. In **Figures 3A–C**, we observed that CPT treatment could down-regulate the expression of NRF2 compared with the control group (CPT group vs. control group, P < 0.05). Although DEB-TACE increased NRF2 expression (P < 0.05), DEB-TACE combined with CPT decreased NRF2 expression compared with the DEB-TACE group (P < 0.05) (**Figures 3A–C**, **S1**). In the CPT group, NRF2 (brownish yellow) was mainly distributed in the cytoplasm. Compared with the control group, NRF2 translocated from the cytoplasm to the nucleus after DEB-TACE treatment, while NRF2 translocation was found decreased after CPT treatment (**Figure 3A**). It was observed that the proportion of nuclear staining in the combined group (16.9%) was significantly lower than that in the TACE group (52.3%). In addition, the reduction of NRF2 downstream genes (GCLM, HO-1 and AKR1C1) and related proteins (NQO1 and HO-1) can be observed from qPCR and Western Blot (**Figures 3C, D**). These results suggest that CPT can inhibit the nuclear translocation of NRF2 and its functional activity after DEB-TACE.

### CPT enhances the efficacy of DEB-TACE on tumors

**Figure 4:**
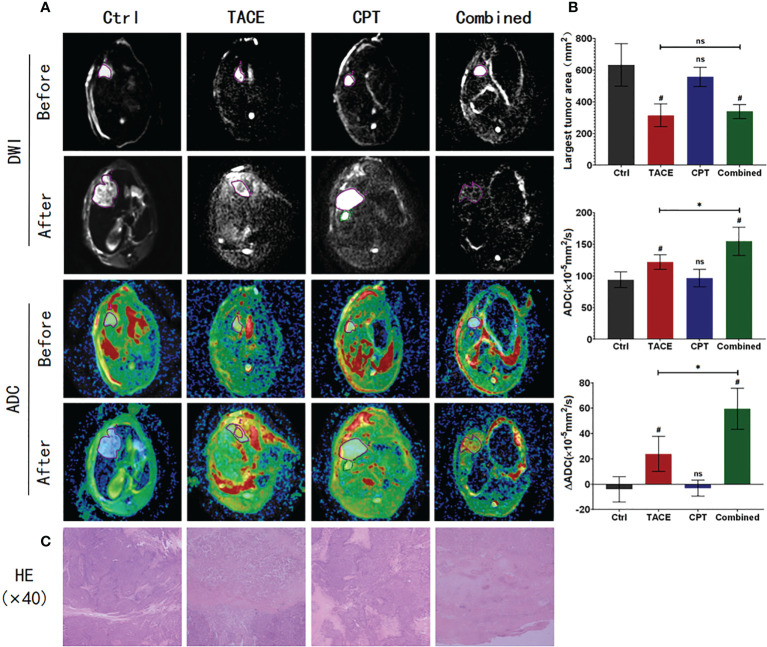
Post-processing and data statistics of magnetic resonance imaging, diffusion-weighted imaging (DWI), and apparent diffusion coefficient (ADC) sequences; high signal DWI, corresponding to low signal ADC, indicates a tumor tissue with restricted diffusion; higher ADC signal indicates lower tumor activity; further observation of tumor necrosis by H&E staining. **(A)** In the after image, the Combined group had more complete low activity tumor areas; areas of hyperactive tumor remained in the TACE group. Diffusion images affected by death in the Ctrl group are shown, and ADC values for this data are not included in the statistics. **(B)** The Combined and TACE groups have smaller maximum tumor areas. The Combined group has lower tumor activity and bigger ΔADC. **(C)** The Combined group had more complete tumor necrosis, whereas the TACE group still had tumor tissue residual at the necrotic margin. ^*^ *P* < 0.05, ^#^ *P* < 0.05, ns *P* > 0.05.

The MRI images were processed, and the maximum interfacial area and ADC and ΔADC values were compared among the four groups of tumors before and 9 days after treatment (**Figure 4A**). The maximum cross-sectional areas 9 days after surgery were 607.8 ± 71.56 mm2, 339.1 ± 53.26 mm2, 557.0 ± 61.27 mm2, and 326.4 ± 56.04 mm2 in the Ctrl, Combined, CPT, and TACE groups, respectively. There was no significant difference between the Combined and TACE groups (P > 0.05), but both had smaller tumor areas than the Ctrl and CPT groups (P < 0.05). There was no significant difference in the tumor cross-sectional area between the Ctrl and CPT groups (P > 0.05). The ADC values 9 days after surgery were 94.13 ± 12.40 (×10–5 mm2/s), 121.9 ± 11.59 (×10–5 mm2/s), 96.75 ± 13.63 (×10–5 mm2/s), and 154.9 ± 22.29 (×10–5 mm2/s) in the Ctrl, TACE, CPT, and Combined groups, respectively. The ADC value of the Combined group was higher than that of the TACE group (P < 0.05), and there was no significant difference between the Ctrl and CPT groups (P > 0.05). The ADC changes were –4 ± 10.1, 23.9 ± 13.8, –3 ± 6.3, and 59.5 ± 16.2 in the Ctrl, TACE, CPT, and Combined groups, respectively (**Figure 4B**). The Combined group had a significantly higher score than the other three groups (P < 0.05).

H&E staining of tumor tissue was performed to observe the degree of tumor necrosis (**Figure 4C**). In the Combined group, necrotic cells were observed in the full field of view, and only a few tumors morphological cells were observed. In the TACE group, a large number of necrotic tumor cells were observed in the center of the tumor. However, residual tumor cells were observed at the edge, and there was no significant difference in the tumor area between the Ctrl and CPT groups.

### CPT did not exacerbate liver damage

**Figure 5:**
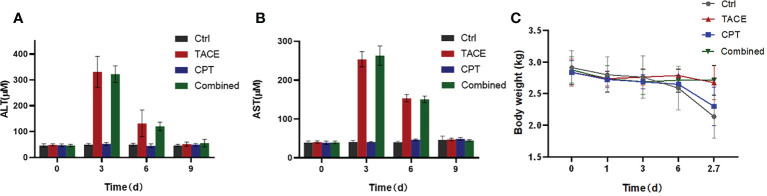
General physiological and biochemical assessment **(A, B)**. Drug-eluting bead transarterial chemoembolization (DEB-TACE) produced a clear image of liver function, which recovered 9 days after surgery. Concomitant use of camptothecin does not significantly impose an additional liver function burden on DEB-TACE. **(C)** With the continuous consumption of the tumor, the body weight of the Ctrl and CPT group continuously decreased.

The effect of CPT on aspartate aminotransferase (AST) and alanine transaminase (ALT) levels was assessed (**Figures 5A, B**). Three days after the intervention, the AST and ALT levels in the TACE and Combined groups increased significantly, and there was no statistically significant difference between the two groups (AST: 253.7 ± 19.45 μm vs. 263.2 ± 25.04 μm, P < 0.05; ALT: 331.1 ± 60.06 μm vs. 322.4 ± 31.97 μm, P < 0.05). On the 6th day, the AST and ALT levels in the CPT and Combined groups significantly decreased. On the 9th day after the operation, the AST and ALT levels in the TACE and Combined groups recovered to a lower level, and there was no statistically significant difference among the four groups (P > 0.05). On the 9th day, the weights of the Ctrl and CPT groups were lower than those of the other two groups, and there was a statistically significant difference (P < 0.05). At each time point, there was no significant difference in body weight between groups A and B (**Figure 5C**).

## Discussion

DEB-TACE inherits the advantages of local chemotherapy of conventional TACE (cTACE), can block blood flow more fully, and can release the drug more persistently. The follow-up of 2218 patients treated with cTACE or DEB-TACE in 14 studies showed that the objective control rate and overall survival time of patients with hepatocellular carcinoma (HCC) treated with TACE were significantly higher than those treated with cTACE (25–27). However, the real-time release dose of DEB-TACE is lower than that of cTACE (5). To a certain extent, the tumor-killing ability of high-concentration chemotherapeutic drugs is lost. In this case, there is a need to improve the utilization of less-dosed chemotherapeutics after DEB-TACE. NRF2 is involved in the development of tumor chemotherapy resistance, and hypoxia caused by adequate embolization of DEB-TACE may also exacerbate NRF2 activation. NRF2 is a suitable inhibitory target for improving the chemotherapeutic utilization of DEB-TACE.

From the perspective of NRF2 detoxification and antioxidant capacity, we explored solutions for drug resistance and attempted to improve chemotherapeutic utilization of DEB-TACE. NRF2 can protect normal cells from DNA damage caused by toxins, such as ROS, thereby inhibiting the activation of proto-oncogenes and damage to tumor suppressor genes. In stark contrast, NRF2 helps tumors defend themselves against adverse factors during their development (13). In particular, the activation of the hyperoxidative state after chemotherapy causes more NRF2 factors to be uncoupled from Kelch-like ECH-associated protein 1 (KEAP1). Subsequently, NRF2 in the cytoplasm enters the nucleus to combine with AREs to activate the expression of antioxidant-related genes (28, 29). Antioxidative processes inhibit the killing effects of inflammation and oxidative stress on tumor cells, ultimately protecting tumor cells and leading to drug resistance. In mouse and cellular experiments, we demonstrated that NRF2 expression is upregulated after tumor chemotherapy (15). From the immunohistochemical results of this experiment, the expression level of NRF2 in the cytoplasm of tumor cells in the TACE group was higher than that of the Ctrl group (P < 0.05), and the NRF2 staining in the nucleus was more obvious. In conclusion, it is theoretically feasible to apply NRF2 inhibitors to inhibit the antioxidant regulation ability of tumor cells after chemotherapy and to attenuate the chemotherapy resistance of tumors.

The use of drugs to increase ROS levels has been proposed to improve tumor therapy efficacy by disrupting the balance between oxidative stress and antioxidant processes within the tumor cells (30). In addition, the combination of ROS inducers and KEAP1-NRF2 system inhibitors can inhibit drug resistance in non-small cell lung cancer (31). Disrupting the above balance, allowing the tumor to lose the regulation of oxidative stress, and enhancing the cytotoxic effect of chemotherapy drugs are new directions to fight chemotherapy resistance and improve the efficacy of chemotherapy.

Our research group discovered and used CPT to inhibit NRF2 expression for the first time and explored the ability of CPT to improve chemotherapy sensitivity by acting on NRF2. CPT and its derivatives have been widely used clinically because of the presence of TOP1 inhibitor. Recent studies have shown that amphiphilic CPT prodrug significantly increases the solubility of CPT (32). We previously demonstrated in vitro cell and mouse experiments that CPT can inhibit NRF2 by inhibiting NRF2 transcription. A lower effective dose of CPT (3 mg/kg/3d) can inhibit the transcriptional activity of NRF2 in tumor-bearing mice after chemotherapy and improve the efficacy of chemotherapeutic drugs (8, 15). Based on the immunohistochemical results of this experiment, after the application of CPT, the expression levels of NRF2 in the Combined groups were lower than that in the TACE groups. Further, CPT inhibited the nuclear translocation of NRF2 and reduced the transcription and protein translation of NRF2 downstream genes.

In this study, DEB-TACE combined with CPT resulted in a better tumor inhibition effect. After treatment, compared with the CPT group, the Combined group had a higher ADC value and a larger ADC change in the tumor area (P < 0.05) and had less residual active tumor at the tissue margin. These results indicate that CPT inhibits the high expression of NRF2 after DEB-TACE, improves the utilization rate of chemotherapeutic drugs, and enhances the efficacy of DEB-TACE. Lower tumor activity inevitably reduces tumor growth. In terms of tumor area 9 days after surgery, no significant difference was found between the TACE and Combined groups, but theoretically, a difference would be observed over a longer observation period. Based on the measurement of AST and ALT levels and body weight at multiple time points, there was no significant difference between the Ctrl and CPT groups and between the TACE and Combined groups (P < 0.05), indicating that the addition of CPT after DEB-TACE does not significantly affect liver function and body weight. This indicates the biosafety of the combination therapy.

In addition to exerting detoxification and antioxidant capacity after treatment, NRF2 also has other pathways that promote tumor development in the absence of treatment. NRF2 accelerates cancer cell proliferation by affecting epidermal growth factor receptor signaling and upregulating anabolism (33, 34). NRF2 can promote epithelial–mesenchymal transition by downregulating E-cadherin expression (35, 36). Mutations in related genes, such as KEAP1 and NRF2, in tumor cells eliminate the interaction between KEAP1 and NRF2 and cause abnormal activation of NRF2 (37, 38). Studies have shown that the incidence rate of KEAP1 or NRF2 mutation in primary liver cancer (HCC) can reach up to 14% (39, 40). Other studies have shown that hepatitis B virus can also stimulate NRF2 activation and upregulate glucose-6-phosphate dehydrogenase (the first rate-limiting enzyme of the pentose phosphate pathway) (41). These phenomena are not directly related to treatment; however, activated NRF2 promotes tumor growth and prepares tumor cells to resist radiation and chemotherapy damage. In theory, chemotherapy-naive tumors will inhibit tumor growth to a certain extent after NRF2 inhibition. The ability of CPT to inhibit TOP1 may also have played a role in this experiment. Compared with the Ctrl group, the CPT group had lower NRF2 expression, but there was no significant difference in the maximum tumor cross-sectional and ADC changes between the CPT and Ctrl groups. This may be due to the fact that lower levels of NRF2 inhibition and lower doses of CPT did not result in macroscopic changes in tumors. Alternatively, the actual difference was small, the sample size of the experiment was small, and no change was detected.

This study has some limitations. As mentioned earlier, this study was not based on a detailed dose optimization process, and the balance between CPT dose and tumor therapy remains to be resolved. We then evaluated the pharmacokinetic and pharmacodynamic characteristics of the combination regimen to optimize dose and efficacy. As the treatment groups were not blinded, the design inherently has the potential for biased assessments. Finally, although both TACE and CPT have been widely and safely used in clinical practice, and the combination therapy in this experiment did not show significant differences in AST and ALT levels, only representative serum AST and ALT tests were performed after combination therapy. However, an in-depth evaluation of the toxicity profile was insufficient.

## Conclusion

CPT suppressed the high expression of NRF2 after DEB-TACE and showed therapeutic benefits in a liver tumor model. The combination of CPT and EPI-loaded microspheres for transarterial chemoembolization is safe and feasible. Future studies should clarify and optimize the pharmacological properties of combination regimen for clinical translation.

## Data availability statement

The raw data supporting the conclusions of this article will be made available by the authors, without undue reservation.

## Ethics statement

The animal study was reviewed and approved by The Laboratory Animal Care and Use Committee of Qianfoshan Hospital of Shandong Province.

## Author contributions

FC and FM designed the studies. FM and YL carried out the study, including data collection and data analysis. QL, HW, and LS performed the data analysis. FM and XL wrote the original draft. FC edited the manuscript. GL and FC supervised. All authors contributed to the article and approved the submitted version.

## Funding

This study was funded by the Natural Science Foundation of Shandong Province (grant no. ZR2019BH041 to FC), the Nature Science Foundation of China (grant no. 81803008 to FC) and the cultivating fund of the First hospital of Shandong First Medical University (grant no. QYPY2021NSFC0616 to FC).

## Acknowledgments

We thank the Experimental Center and Medical Imaging Department of Qianfoshan Hospital of Shandong Province for providing relevant consultation and instrument support.

## Conflict of interest

The authors declare that the research was conducted in the absence of any commercial or financial relationships that could be construed as a potential conflict of interest.

The reviewer GZ declared a shared parent affiliation with the author QL to the handling editor at the time of review.

## Publisher’s note

All claims expressed in this article are solely those of the authors and do not necessarily represent those of their affiliated organizations, or those of the publisher, the editors and the reviewers. Any product that may be evaluated in this article, or claim that may be made by its manufacturer, is not guaranteed or endorsed by the publisher.
