# Supplementary figures and images for: Experimental study of camptothecin combined with drug-eluting bead transarterial chemoembolization in the rabbit VX2 liver tumor model
Source: Front Oncol. 2022 Oct 10;12:906971. doi: 10.3389/fonc.2022.906971 (PMC9590107; doi:10.3389/fonc.2022.906971)

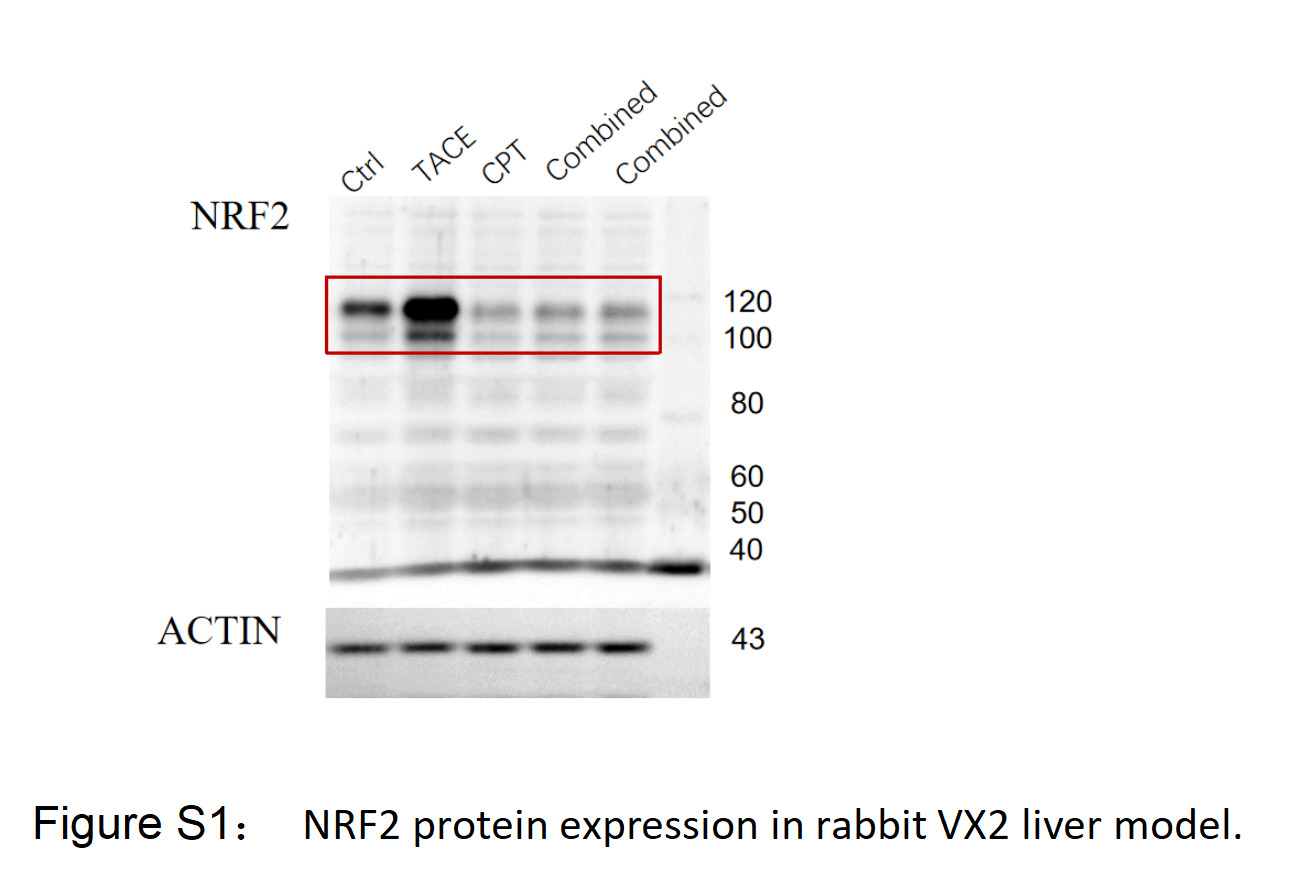

Supplement: Supplementary file 1 [file Image_1.jpg]
